# Supplementary material for: Combination of Plasma-Based Metabolomics and Machine Learning Algorithm Provides a Novel Diagnostic Strategy for Malignant Mesothelioma
Source: Diagnostics (Basel). 2021 Jul 16;11(7):1281. doi: 10.3390/diagnostics11071281 (PMC8304303; doi:10.3390/diagnostics11071281)
Supplement: Supplementary file 1 [file diagnostics-11-01281-s001.zip › diagnostics-1280990-supplementary.pdf]

Table S1. AUC of ROC; sensitivity and specificity at optimal cut-off value for each metabolite and the machine learning model.

Model	Train set		
	**AUC**	**Specificity**	**Sensitivity**
Taurocholic acid	0.8421	0.6667	0.9474
Uracil	0.8399	0.8333	0.8421
Biliverdin	0.8289	0.6667	0.9474
Histidine	0.8180	0.8750	0.6842
Tauroursodeoxycholic acid	0.8048	0.6250	0.8947
Pyrroline hydroxycarboxylic acid	0.8026	1.0000	0.6316
Phenylalanine	0.8004	0.9583	0.5789
Random forest model (RF)	1.0000	1.0000	1.0000
